# Conversion of Phenol and Lignin as Components of Renewable Raw Materials on Pt and Ru-Supported Catalysts

**DOI:** 10.3390/molecules27051494

**Published:** 2022-02-23

**Authors:** Aleksey E. Koklin, Nataliia A. Bobrova, Tatiana V. Bogdan, Igor I. Mishanin, Viktor I. Bogdan

**Affiliations:** 1 N.D. Zelinsky Institute of Organic Chemistry, Russian Academy of Sciences, Leninsky Prospect, 47, 119991 Moscow, Russia; nat.bobrowa@yandex.ru (N.A.B.); chemist2014@yandex.ru (T.V.B.); arnochem@yandex.ru (I.I.M.); vibogdan@gmail.com (V.I.B.); 2 Chemistry Department, Lomonosov Moscow State University, Leninskie Gory, 1, bldg. 3, 119991 Moscow, Russia

**Keywords:** lignin, lignosulfonate, phenol, hydrogenation, Pt, Ru-supported catalysts

## Abstract

Hydrogenation of phenol in aqueous solutions on Pt-Ni/SiO_2_, Pt-Ni-Cr/Al_2_O_3_, Pt/C, and Ru/C catalysts was studied at temperatures of 150–250 °C and pressures of 40–80 bar. The possibility of hydrogenation of hydrolysis lignin in an aqueous medium in the presence of a Ru/C catalyst is shown. The conversion of hydrolysis lignin and water-soluble sodium lignosulfonate occurs with the formation of a complex mixture of monomeric products: a number of phenols, products of their catalytic hydrogenation (cyclohexanol and cyclohexanone), and hydrogenolysis products (cyclic and aliphatic C_2_–C_7_ hydrocarbons).

## 1. Introduction

Biomass is considered as an alternative to fossil raw materials, both for the production of motor fuel components and for the synthesis of a wide range of valuable chemical compounds [1]. Existing technologies for processing biomass are based on the delignification of wood in the process of paper production. In addition to combustible gas products, a complex viscous mixture contains a large amount of phenolic compounds and water. A distinctive feature of such a mixture, in comparison with traditional oil, is the high oxygen content, which negatively affects its calorific value and leads to the need for additional stages of processing. One of the main methods of removing oxygen (and other heteroatoms) from organic compounds is catalytic hydrotreatment.

Hydrodeoxygenation of lignocellulose components is one of the most effective ways to remove oxygen from oxygenates. Hydrogenolysis is performed on aluminum oxide-supported Ni-Mo and Co-Mo sulfides [2,3,4,5,6,7] or bifunctional catalysts [8,9]. The latter catalytic systems combine two active catalytic functions: supported metal catalysts (noble metals or Ni) and acids (CH_3_COOH, H_3_PO_4_) or Brønsted solid acids (Nafion). Despite a large amount of research in this area, the valorization of lignin materials by conversion to value-added chemical and fine chemical products is a highly important task and remains a considerable challenge. The aim of this work is the heterogeneous catalytic hydrogenation of phenol and aqueous solutions of lignin-containing substrates on metal-supported catalysts.

## 2. Results and Discussion

According to the results shown in Table 1, phenol hydrogenation proceeds more efficiently on the Ru/C catalyst, compared to Pt-containing catalysts. Promotion of the Pt-Ni catalyst with chromium does not lead to any significant changes in the total conversion of phenol.

The acidity of the aqueous solution has a significant effect on the distribution of the reaction products. An increase in acidity leads to an almost complete conversion of phenol at 150 °C and to a significant increase in the yield of aliphatic and cyclic C_2_–C_6_ hydrocarbons, which are formed due to the acceleration of the processes of dehydration of cyclic oxygenates and subsequent destructive hydrogenation.

The Ru/C system demonstrated the highest activity among the catalysts of the studied series: the conversion of phenol is close to 100% already at 150 °C. A distinctive feature of this catalyst is the high selectivity of cyclohexanol formation exceeding 90%; however, an increase in the acidity of the substrate does not lead to such a significant increase in the yield of light products as was observed in the case of the Pt-Ni/SiO_2_ catalyst. Cyclohexanone is formed only in small amounts for all the conditions studied in this work. Other products were aliphatic and cyclic hydrocarbons C_2_–C_6_. The yield of the hydrocarbon addition of phosphoric acid led to an increase in the yield of hydrocarbons from 13% to 93% at 200 °C (in an autoclave). It should be noted that the formation of aromatic hydrocarbons was not detected under the conditions studied, that is, the cleavage of the hydroxyl group of phenol while preserving the aromatic ring was not observed. Table 2 shows the distribution of phenol hydrogenolysis products formed on the Ru/C catalyst. Regardless of the acidity of the medium and the type of the process, the main hydrocarbon product was cyclohexane. The addition of phosphoric acid to the phenol solution contributes to a significant increase in the yield of aliphatic and cyclic C_2_–C_6_ hydrocarbons due to the acceleration of the dehydration of cyclohexanol into cyclohexene with its subsequent hydrogenation and destructive hydrogenolysis into hydrocarbons. In the absence of acid, the dehydration of cyclohexanol occurs under the action of hydrogen ions present in the solution due to the dissociation of water molecules. With increasing temperature, the degree of water dissociation increases (pKw = 11.2 at 250 °C [10]): this may be associated with a significantly higher yield of hydrocarbons at 250 °C.

The results obtained in the study of phenol hydrogenation showed that the Ru/C catalyst can be considered as a very promising catalyst for the hydrogenation of lignin-containing materials in an aqueous medium. The conversion of hydrolytic lignin (LH) and sodium lignosulfonate (LS) was further studied using this catalyst. Also, the efficiency of the ruthenium catalyst was compared with the commercially available platinum catalyst 10 wt. % Pt/C (Aldrich). It is noteworthy that the activity of low-loaded Pt, Pt-Ni, and Pt-Ni-Cr catalysts that exhibited a good performance in phenol hydrogenation was negligible in the case of the lignin hydroconversion.

**Figure 1:**
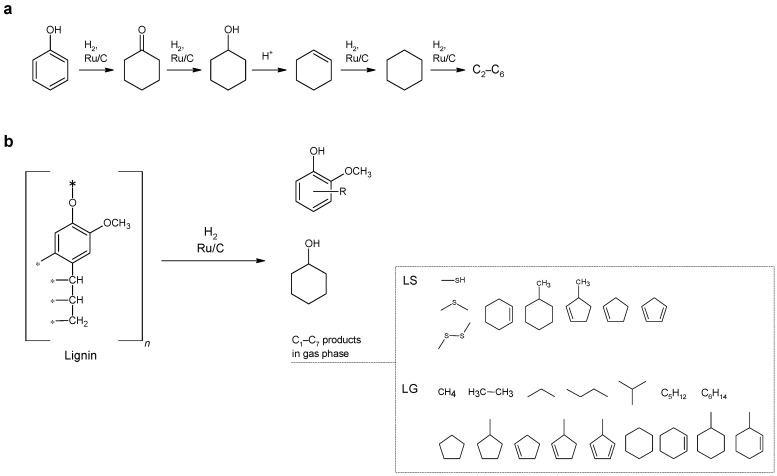
Catalytic conversion of (**a**) phenol and (**b**) lignin.

Analysis of the composition of the products (see Figure 1, Table 3) suggests that the primary reaction of aqueous solutions of lignin-containing materials is hydrolysis with the formation of mono-, di-, and oligophenols. Then, the catalytic transformations of phenols occur with the participation of hydrogen: hydrogenation of the benzene ring into cyclohexanol and cyclohexanone and hydrogenolysis of C-O and C-C bonds. In the course of the catalytic hydrogenation of lignin, a number of light aliphatic and cyclic hydrocarbons C_2_–C_7_ were found in the gas phase. In the case of hydrogenation of sodium lignosulfonate, sulfur-containing organic compounds (methanethiol, dimethyl sulfide, dimethyl disulfide) are also formed.

**Figure 2:**
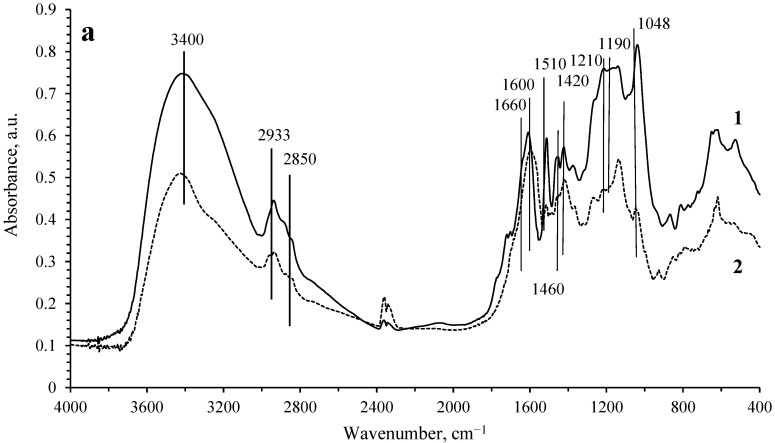

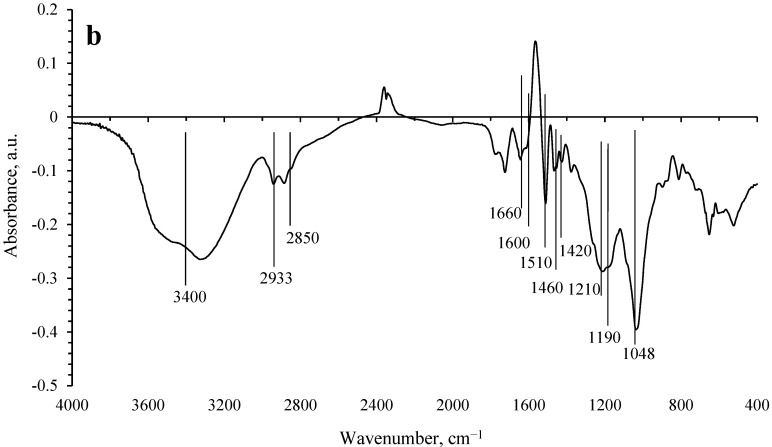
(**a**) IR spectra of sodium lignosulfonate (**1**) before and (**2**) after hydrogenation (250 °C, Ru/C). (**b**) The difference IR spectrum of lignosulfonate after and before hydrogenation.

The transformation of lignosulfonate in the process of hydrogenation was studied by IR spectroscopy. The IR spectra of the initial LS, as well as the spectra of the solid phase remaining after the hydrogenation process on the Ru/C catalyst, are shown in Figure 2. Based on the literature data [11], the observed absorption bands can be attributed as follows. The wide band at 3400 cm^−1^ is assigned to the stretching vibrations of OH groups in phenolic and aliphatic structures. The bands at 2933 and 1460 cm^−1^ correspond to the antisymmetric stretching and bending vibrations of C-H in CH_3_ and CH_2_ groups. The band at 2850 cm^−1^ is attributed to the C-H symmetric stretching vibrations in CH_2_ groups or C-H stretching vibrations in CH_3_O groups. The band at 1660 cm^−1^ belongs to the stretching vibrations of C=O groups. The bands at 1600, 1510, and 1420 cm^−1^ are assigned to the vibrations of the aromatic ring. The band at 1210 cm^−1^ is attributed to the stretching vibrations of the C-O bond in guaiacyl fragments. The bands at 1190 and 1048 cm^−1^ can be assigned to vibrations in the SO_3_^2−^ groups.

Analysis of the difference spectrum of the solid phase before and after hydrogenation (Figure 2b) shows that hydrogenation leads to a significant decrease in the concentration of OH groups. The intensity of the absorption bands belonging to the SO_3_^2−^ groups is also significantly reduced. In turn, the decrease in the intensity of the absorption bands related to CH_2_ and CH_3_ groups is significantly lower than those for OH and SO_3_^2−^ groups. Even less noticeable are the changes in the intensities of the absorption bands in the IR spectra related to the vibrations of the aromatic ring. Based on the analysis of IR spectroscopy data, it can be concluded that the aromaticity of the lignin polymer framework is preserved in general. In the process of hydrogenation, transformations mainly occur with the participation of hydroxyl groups of lignin, as well as sulfite groups, that is, the process of lignin hydrotreating occurs. These transformations can lead to the cleavage of fragments containing a single aromatic ring, which subsequently undergoes hydrogenation. At the same time, hydrogenation of aromatic rings that are part of the lignin polymer framework is not observed.

The data on the elemental analysis of hydrolysis lignin and solid residue obtained after extraction are presented in Table 4. These data clearly show a much lower content of oxygenated moieties in the solid residue. The distribution of the gas products formed on Ru/C and Pt/C catalysts in the hydrogenation of water-soluble lignin in an aqueous medium at 250 °C is given in Table 5. The overall yield of gas products is significantly higher on the Ru/C catalyst compared to the Pt/C catalyst, whereas methane predominates among the hydrocarbons formed on both catalysts.

**Figure 3:**
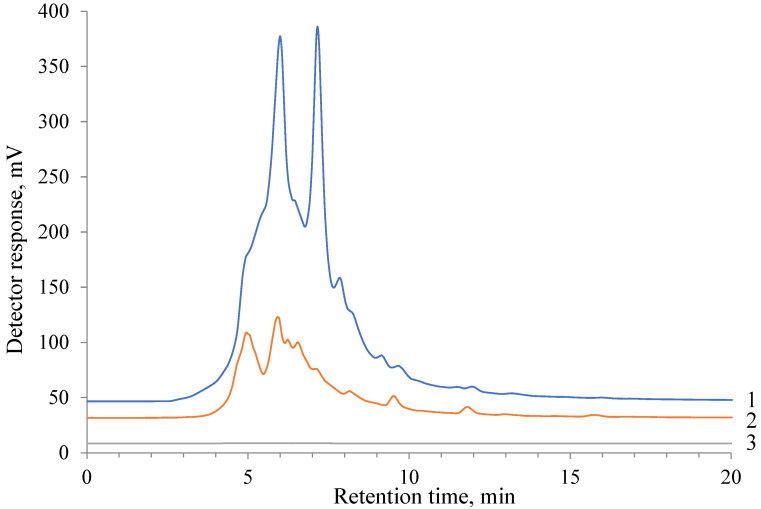
HPLC chromatograms of water-soluble lignin (**1**) before and after hydrogenation on (**2**) Pt/C and (**3**) Ru/C catalysts at 250 °C.

The data on the concentrations of light-weight products identified by GC-MS in the aqueous medium before and after hydrogenation at 250 °C for both catalysts are presented in Table 6 in comparison with the composition of the water-soluble lignin sample. Cyclohexanol predominates on the Ru/C catalyst, whereas the Pt/C catalyst provides a more uniform distribution of liquid products, including phenols, diphenols, and alcohols. Depolymerization of hydrolytic lignin via the interaction of a solid substance with water leads to the formation of an aqueous solution of oligomeric and monomeric fragments of the initial lignin. Analysis of the obtained initial water-soluble lignin and hydrogenated products obtained using Pt/C and Ru/C catalysts at 250 °C was analyzed by HPLC (Figure 3). It can be seen that lignophenols of the initial aqueous solution, the total amount of which is detected by the presence of the aromatic structures in UV at 290 nm, significantly decrease on the Pt/C catalyst and completely disappear during hydrogenation on the Ru/C catalyst, other parameters being equal. Consequently, the aromatic structure of the phenols of the oligomeric fragments of lignin and its monomeric units undergo exhaustive hydrogenation on the Ru/C catalyst at 250 °C. This result confirms the data of the GC-MS analysis in Table 6. 

## 3. Materials and Methods

Table 7 shows the list of catalysts studied in this work. Catalysts No. 1–2 are commercial catalysts produced by the Redkino Catalyst Plant, Russia. Catalyst No. 3 is a commercial catalyst (Aldrich, Steinheim, Germany). Catalyst No. 4 is a ruthenium catalyst prepared by the incipient wetness impregnation method. Commercial carbon material Sibunit [12], produced by the Center of New Chemical Technologies of the Boreskov Institute of Catalysis of the Russian Academy of Sciences (Omsk, Russia) with average granule diameters of 1.5–1.8 mm was used as the support. Before use, Sibunite was subjected to oxidative treatment according to the previously described method [13]. Sibunit was impregnated with an aqueous solution of ruthenium hydroxychloride (Ru(OH)Cl_3_), then the sample was dried in air at 80 °C and reduced in a hydrogen flow at 400 °C prior to the catalytic tests.

The textural characteristics of the samples were studied using low-temperature nitrogen adsorption at 77 K on an Autosorb iQ gas sorption analyzer (Quantachrome inst., Boynton Beach, FL, USA). The specific surface area of the samples was calculated by the BET method.

For this study, phenol (chemically pure grade) and lignin-containing substrates were used: technical water-soluble sodium lignosulfonate (LS, “Solikamskbumprom”, Solikamsk, Russia) and water-insoluble hydrolysis lignin (LH, in the form of an enterosorbent “Polyphepan”, Russia).

Hydrogenation of phenol was carried out in a flow-type reactor in the temperature range 150–250 °C at a pressure of 75 bar. The volume of the loaded catalyst was 0.5 cm^3^, the remaining free volume of the reactor was filled with quartz chips. Activation of the catalyst was carried out in a hydrogen flow at a temperature of 320 °C immediately before the experiment. The reaction was carried out in an aqueous solution with a phenol concentration of 10 g/L. The flow rate of the solution was 1 mL/min, the hydrogen gas flow rate was 40 cm^3^/min (molar ratio phenol: H_2_ = 1:16). The sample for analysis was taken 60 min after the start of the phenol supply. In a number of experiments, orthophosphoric acid H_3_PO_4_ was added to an aqueous solution of phenol in an amount of 0.5 wt. % to introduce acidity.

Moreover, hydrogenation of phenol was studied in a batch reactor using the Ru/C catalyst. The experiments were carried out in a 100 mL autoclave (Reaction Engineering, Anyang, Korea). The loading of the Ru/C catalyst was 0.05 g. Phenol (0.60 g) and 60 mL of distilled water were placed in the reactor. The hydrogen pressure was 45 bar at room temperature, and after heating to a reaction temperature of 150, 200, or 250 °C, the pressure increased to 60, 70, and 80 bar, respectively. The reaction was carried out by stirring with a mechanical amount of LS loaded was 0.60 g, and the amount of Ru/C catalyst was 0.10 g. Catalytic hydrogenation of LS was carried out at a temperature of 250 °C, a hydrogen pressure of 35 bar (at room temperature), a stirring speed of 600 rpm, and a reaction time of 4 h. Hydrogenation of hydrolysis lignin was carried out by using a slightly different method. First, LH was extracted with water at 250 °C. In a 600 mL autoclave (Parr Instruments Co., Moline, IL, USA), 15 g of hydrolysis lignin and 350 mL of distilled water were placed. The autoclave was purged with argon and then filled with argon until a pressure of 40 bar was reached. Then, LH was held at a temperature of 250 °C for 4 h with stirring at 600 rpm. The extract was filtered on a Buchner funnel. The undissolved residue was dried in an oven at 50 °C. The weight of the dried undissolved residue was about 11 g. Therefore, the concentration of extracted lignin was 11.4 mg/mL. At the second step, the resulting solution with water-soluble hydrolysis lignin was loaded in an autoclave. The Ru/C or Pt/C catalyst loading was 1 g. The catalysts were activated in hydrogen at 400 °C prior to the catalytic tests. The hydrogenation was performed at 250 °C, the pressure of H_2_ was 40 bar at room temperature, and the stirring speed was 600 rpm.

The products were analyzed by gas chromatography using a Chromatec Crystal 5000 chromatograph with a Thermo TR-5ms capillary column and CaA and Porapak Q packed columns (Yoshkar-Ola, Russia). The concentration of components in the aqueous phase was determined using butanol-1 and phenol as internal standards in the experiments with phenol and LH/LS, respectively. The components in the product mixtures were identified using a Thermo Focus GC/DSQ II chromato-mass-spectrometer with a Thermo TR-5ms column.

The water-soluble products of lignin depolymerization were analyzed by HPLC using a model 481 UV detector (Waters, Milford, MA, USA) at 290 nm and Phenomenex Luna C18(2) column a mixture of water and acetonitrile (1:1) was used as an eluent with a flow rate of 0.5 mL/min.

IR spectra (with KBr) were recorded using a Bruker Alpha-T IR spectrometer (Bruker optics, Ettlingen, Germany)in the transmission mode with a resolution of 4 cm^−1^.

## 4. Conclusions

The obtained regularities of the catalytic conversion process of phenol and lignin indicate the common features of reactions and mechanisms of the reductive transformation of phenolic structures with the formation of a wide range of hydrocarbons. Studies have shown that the Ru/C catalyst is active in the hydrogenation of both phenol and lignin. Using the example of phenol hydrogenation, it was shown that an increase in the acidity of the medium makes it possible to intensify the process of hydrotreating reaction products to remove organic compounds containing heteroatoms.

## Figures and Tables

**Table 1:** Hydrogenation of phenol in an aqueous medium in continuous and periodic modes.

Catalyst	Medium ^1^	*T*, °C	*P*, atm	Conversion, %	Selectivity, %		
					Cyclohexanol	Cyclohexanone	Hydrocarbons C_2_–C_6_
Continuous mode (flow reactor)							
Pt-Ni/SiO_2_	H_2_O	150	75	51	86	6	8
Pt-Ni/SiO_2_	H_2_O-H_3_PO_4_	150	75	88	58	16	26
Pt-Ni-Cr/Al_2_O_3_	H_2_O	150	75	47	81	19	-
Ru/C	H_2_O	150	75	98	94	1	5
Ru/C	H_2_O-H_3_PO_4_	150	75	100	89	0	11
Periodic mode (autoclave) ^2^							
Ru/C	H_2_O	150	60	100	92	0	8
Ru/C	H_2_O-H_3_PO_4_	150	60	100	81	1	18
Ru/C	H_2_O	200	70	100	86	1	13
Ru/C	H_2_O-H_3_PO_4_	200	70	100	7	0	93
Ru/C	H_2_O	250	80	100	48	1	51

^1^ Distilled water (H_2_O) or 0.5 wt. % orthophosphoric acid solution (H_2_O-H_3_PO_4_). ^2^ Pressure of hydrogen was 45 bar at room temperature.

**Table 2:** Distribution of gaseous products (vol. %) in the hydrogenation of phenol in an aqueous medium on a Ru/C catalyst at 200 °C and 70 atm H_2_ (in an autoclave).

Products	H_2_O	H_2_O-H_3_PO_4_
C_2_H_6_	17.5	5.5
C_3_H_8_	1.1	0.6
C_4_H_10_	2.1	0.8
C_5_H_12_	8.5	2.2
C_6_H_14_	1.5	0.8
Cyclopentane, methylcyclopentane	0.9	0.4
Cyclohexane	66.7	76.2
Cyclohexene	1.7	13.5

**Table 3:** Products of hydrolytic lignin (LH) and sodium lignosulfonate (LS) conversion on the Ru/C catalyst at 250 °C.

Substrate	Yield, wt. %		
	Solid Residue	Gas Products	Water-Soluble Products
LH	73	7	20
LS	29	< 1	71

**Table 4:** Elemental analysis results of hydrolysis lignin and solid residue after extraction.

Sample	C, %	H, %	O, % ^1^
Hydrolysis lignin ^2^	58.1	6.0	35.9
Solid residue	68.1	5.9	26.0

^1^ Calculated by difference. ^2^ The sample contains no nitrogen and no sulfur; the ash content is about 1.5%.

**Table 5:** Distribution of gaseous products (vol. %) in the hydrogenation of water-soluble lignin in an aqueous medium on Ru/C and Pt/C catalysts at 250 °C.

Catalyst	Ru/C	Pt/C
Yield of hydrocarbons, % ^1^	25.6	1.4
CH_4_	96.6	61.3
C_2_–C_5_ ^2^	2.5	12.4
Cyclopentane	0.3	6.7
Methylcyclopentane	0.1	12.0
Cyclohexane	0.2	3.7
Others	0.3	3.9

^1^ Yield of hydrocarbons = (amount of C in gas products)/(amount of C in the aqueous medium at the initial moment) × 100%. ^2^ Mainly butanes and pentanes.

**Table 6:** Concentration of lightweight products identified by GC-MS in aqueous medium before and after hydrogenation at 250 °C.

Compound		Concentration, μg/mL		
Structural Formula	Name	Water-Soluble Lignin	Ru/C	Pt/C
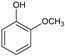	o-Methoxyphenol (Guaiacol)	50.36	1.95	7.26
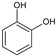	2-Hydroxyphenol (Catechol)	4.39		
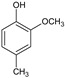	2-Methoxy-4-methyl-phenol			6.07
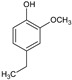	2-Methoxy-4-ethyl-phenol	1.73		8.71
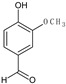	4-Hydroxy-3-methoxybenzaldehyde (Vanillin)	6.28		
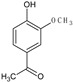	1-(4-Hydroxy-3-methoxyphenyl)ethan-1-one	3.08		
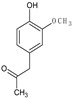	1-(4-Hydroxy-3-methoxyphenyl)propane-2-one	6.58		
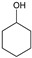	Cyclohexanol		38.63	
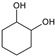	1,2-Dihydroxycyclohexane		4.36	10.17
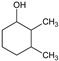	2,3-Dimethyl-cyclohexanol		3.42	
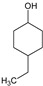	4-Ethylcyclohexanol		3.68	

**Table 7:** Catalysts studied.

No.	Catalyst	Composition, wt. %	Carrier	*S*_BET_, m^2^/g	Bulk Density, g/cm^3^
1	Pt-Ni/SiO_2_	0.8%Pt -3%Ni/SiO_2_	Silica gel	n/d	0.66
2	Pt-Ni-Cr/Al_2_O_3_	0.7%Pt -3%Ni -1.5%Cr/Al_2_O_3_	γ-Al_2_O_3_	n/d	0.67
3	Pt/C	10%Pt/C	activated carbon	760	0.42
4	Ru/C	10%Ru/C	Sibunit	220	0.71

n/d—no data.

## Data Availability

Not applicable.

## Sample Availability

Samples of the catalysts are available from the authors.
